# A Conserved N-Terminal Di-Arginine Motif Stabilizes Plant DGAT1 and Modulates Lipid Droplet Organization

**DOI:** 10.3390/ijms26157406

**Published:** 2025-07-31

**Authors:** Somrutai Winichayakul, Hong Xue, Nick Roberts

**Affiliations:** Resilient Agriculture, AgResearch Grasslands, Palmerston North 4442, New Zealand; hong.xue@agresearch.co.nz (H.X.); nick.roberts@agresearch.co.nz (N.R.)

**Keywords:** DGAT1, oleosin, di-arginine motif, Camelina, yeast, lipid droplets, transmembrane protein

## Abstract

Diacylglycerol-O-acyltransferase 1 (DGAT1, EC 2.3.1.20) is a pivotal enzyme in plant triacylglycerol (TAG) biosynthesis. Previous work identified conserved di-arginine (R) motifs (R-R, R-X-R, and R-X-X-R) in its N-terminal cytoplasmic acyl-CoA binding domain. To elucidate their functional significance, we engineered R-rich sequences in the N-termini of *Tropaeolum majus* and *Zea mays* DGAT1s. Comparative analysis with their respective non-mutant constructs showed that deleting or substituting R with glycine in the N-terminal region of DGAT1 markedly reduced lipid accumulation in both *Camelina sativa* seeds and *Saccharomyces cerevisiae* cells. Immunofluorescence imaging revealed co-localization of non-mutant and R-substituted DGAT1 with lipid droplets (LDs). However, disruption of an N-terminal di-R motif destabilizes DGAT1, alters LD organization, and impairs recombinant oleosin retention on LDs. Further evidence suggests that the di-R motif mediates DGAT1 retrieval from LDs to the endoplasmic reticulum (ER), implicating its role in dynamic LD–ER protein trafficking. These findings establish the conserved di-R motifs as important regulators of DGAT1 function and LD dynamics, offering insights for the engineering of oil content in diverse biological systems.

## 1. Introduction

Plant oil is a valuable commodity, not only due to its extensive use in the food industry and as an ingredient in animal feed but also because of its versatile applications in biofuels, nutraceuticals, and various industrial products. Within the plant itself, oil plays a crucial role in facilitating essential metabolic processes, particularly during seed germination and early plant growth stages, which are vital for overall development. Given its significance, there is a growing research interest in the field of plant biotechnology aimed at enhancing plant oil production and ensuring a more sustainable supply [1,2,3].

Although liquid biofuels show great promise, the practicality of utilizing biological materials is limited by competing uses and their availability in sufficient quantities. As a result, numerous research groups are focused on engineering plants and microorganisms to address these challenges, particularly by enhancing the accumulation of triacylglycerol (TAG) in vegetative tissues, as well as oleaginous yeasts and bacteria [4,5,6]. TAG is a neutral lipid with twice the energy density of cellulose and can be converted into biodiesel, a high-energy-density biofuel with a simple and efficient manufacturing process. Thus far, efforts to engineer TAG accumulation in leaves have resulted in a significant increase, ranging from 1 to 108 times, compared to the wild type (WT). These improvements have been achieved through various integrated strategies, including overexpression of seed development transcription factors such as LEAFY COTYLEDON 1 & 2 and WRINKLED 1 [7,8,9]; silencing of ADP–glucose pyrophosphorylase, a key gene involved in starch biosynthesis [10]; mutation of CGI-58, a regulator of neutral lipid accumulation [11]; upregulation of TAG-synthesizing enzyme diacylglycerol-O-acyltransferase (DGAT, EC 2.3.1.20) in both plants and yeast [12,13,14]; and prevention of TAG catabolism through the modulation of genes such as OLEOSIN and SUGAR-DEPENDENT1 LIPASES, which is particularly crucial in non-oleaginous tissues and throughout various developmental stages [6,10,15,16].

Improving the yield and quality of TAG in eukaryotes is a challenging task. Since diacylglycerol occupies the branch point between TAG and membrane lipid biosynthesis, DGAT likely plays a key role in regulating TAG formation within the glycerolipid synthesis pathway [17]. Despite being in a rate-limiting step, DGAT exhibits the lowest specific activity among the enzymes in the Kennedy pathway and is often considered a ‘bottleneck’ in TAG synthesis. DGAT proteins can be classified into three distinct families. The first family, known as DGAT1s, shares similarities with acyl-coenzyme A:cholesterol acyltransferases, belonging to the membrane-bound O-acyltransferase group [18]. The second family, DGAT2s, is unrelated to DGAT1 and falls under the category of monoacylglycerol acyltransferases and wax ester synthases [19]. Although their functions appear to be relatively diverse, both integral membrane proteins, DGAT1s and DGAT2s, can catalyze the esterification of long-chain fatty acids to nascent diglycerides [20,21,22,23]. The third family, DGAT3s, first identified in plants, comprises soluble acyltransferase contributing to acyl exchanges between TAG and the cytosolic acyl-CoA pool, with a substrate preference for fatty acids (FA) 18:2 and 18:3 [24,25].

DGAT1s are typically responsible for TAG synthesis in seeds and senescing leaves [17,26,27,28]. Previous attempts to enhance DGAT1 through biotechnological methods have yielded limited success. For instance, Nykiforuk et al. (2002) reported a 50% reduction in activity when truncating the N-terminus of *Brassica napus* DGAT1 [29]. McFie et al. (2010) observed increased specific activity upon N-terminal truncation of mouse DGAT1 but also noted a significant decline in the accumulated protein levels [30]. Xu et al. (2008) identified a consensus sequence (X-L-X-K-X-X-S197-X-X-X-V) within *Tropaeolum majus* (garden nasturtium) DGAT1 sequences (*Tm* DGAT1), resembling a targeting motif commonly found in members of the SNF1-related protein kinase 1 (SnRK1) family, with serine being the residue for phosphorylation [31]. SnRK1 proteins are a class of serine/threonine protein kinases increasingly implicated in the global regulation of carbon metabolism in plants, including the phosphorylation-mediated inactivation of sucrose phosphate synthase [32]. Through site-directed mutagenesis in six putative functional regions/motifs of Tm DGAT1, Xu et al. (2008) discovered that mutating a serine residue to alanine (S197A) in the putative SnRK1 target site resulted in a 38–80% increase in DGAT1 activity [31]. Moreover, overexpression of the mutated Tm DGAT1 in Arabidopsis led to a 20–50% enhancement in oil content per seed.

**Figure 1:**
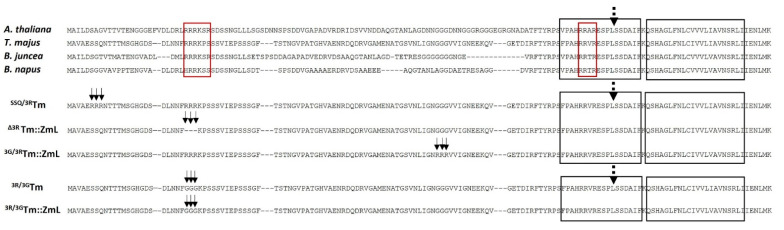
The alignment shows partial N-terminal cytoplasmic sequences (121 residues) of DGAT1 from Brassicales species, including *A. thaliana* (total protein: 522 residues, 9 TMDs), *T. majus* (total protein: 520 residues, 9 TMDs), *B. juncea*, and *B. napus*. Red boxes indicate the conserved di-arginine motif (R-R, R-X-R or R-X-X-R-) near the N-terminus and a second motif (RR or R-R-X-R) in the acyl Co-A binding region. The left-hand black box represents the acyl-CoA binding domain [29], while the right-hand box represents the predicted first transmembrane domain [30]. The dashed arrow indicates the engineered site utilized to generate chimeric Tm::ZmL DGAT1 [35]. The solid arrows indicate the site-specific mutation. For expression in *C. sativa*, the following mutants were used: ^SSQ/3R^Tm, representing the substituted S6R, S7R, and S8R mutant of Tm; ^∆3R^Tm::ZmL, representing the deleted R25, R26, and R27 mutant of Tm::ZmL; ^3G/3R^Tm::ZmL, representing the substituted G78R, G79R, and G80R mutant of Tm::ZmL. For expression in *S. cerevisiae*, the following mutants were used: ^3R/3G^Tm and ^3R/3G^Tm::ZmL, which represent the substituted R25G, R26G, and R27G mutant of Tm and Tm::ZmL, respectively. Accession numbers for the mentioned species are as follows: *A. thaliana*, NP_179535; *T. majus*, AAM03340; *B. juncea*, AAY40785; *B. napus*, AAD45536; *Tm::ZmL*, ON959593.

N-terminal deletion of DGAT or the generation of chimeric DGATs by combining monocotyledonous and dicotyledonous DGAT peptide sequences has shown significant increases in FA content in yeast and Camelina seeds [33,34,35]. The authors have proposed potential roles for conserved di-arginine (R) motifs (R-R, R-X-R, and R-X-X-R) found in the N terminus of the cytoplasmic acyl-CoA binding domain of DGAT1, where X represents any amino acid residue. Two key conserved regions have been characterized: a first di-arginine motif (R-R, R-X-R, or R-X-X-R) located near the N terminus and a second motif (R-R or R-R-X-R) positioned within the acyl-CoA binding region (Figure 1, red box). The di-R motifs have been demonstrated to be associated with various functions, such as targeting of Arabidopsis glucosidase 1 membrane proteins to the endoplasmic reticulum (ER) [36] and determining the topology of paramyxovirus hemagglutinin–neuraminidase [37]. Teasdale and Jackson (1996) demonstrated that di-lysine and di-R motifs located near the terminus of a cytoplasmic domain are involved in retrieving ER membrane proteins from the Golgi apparatus and the ER–Golgi intermediate [38]. Moreover, di-R has been reported to play a role in the assembly of heteromultimeric membrane proteins [39,40].

In this study, we provide evidence for the functional role of the cytoplasmic N-terminal di-R motif in plant DGAT1s, specifically in the stability of proteins, with or without the presence of lipid-associated protein oleosins. Furthermore, considering the partitioning of plant DGAT1 to lipid droplets (LDs) [35], we also investigated the potential involvement of di-R motifs as ER retrieval signals from the LDs.

## 2. Results

### 2.1. A Modified N-Terminal di-R Motif of DGAT1 Influences Lipid Content in C. sativa Seeds

The majority of angiosperm DGAT1 enzymes feature diverse di-R motifs within the variable regions of their cytoplasmic N terminus, as reported by Winichayakul et al. (2022) [35]. Sequence alignment of DGAT1 from the *Brassicales* order—including *Arabidopsis thaliana* (NP_179535; AAM03340), *Tropaeolum majus* (AAM03340), *Brassica juncea* (AAY40785), and *Brassica napus* (AAD45536)—reveals two conserved di-R motifs: one near the N terminus (R-R-R or R-X-X-R-R) and another within the N-terminal acyl-CoA binding region (R-R-X-R) (Figure 1). To explore potential roles of the cytoplasmic N-terminal di-R motifs in DGAT1, we overexpressed the full-length *Tm* DGAT1, a chimera between *Tm* DGAT1 and *Zea mays* DGAT1 long-form (*Tm::ZmL*), and three site-specific di-R mutants (^SSQ/3R^*Tm*, ^Δ3R^*Tm::ZmL*, and ^3G/3R^*Tm::ZmL*) in developing seeds of *C. sativa* (Figure 1). Chimera Tm::ZmL was previously reported to significantly increase lipid content (mg/seed) and seed size [35], prompting its continued use in this study alongside full-length Tm DGAT1. The specific di-R mutations included ^SSQ/3R^*Tm* (substitution of S6R, S7R, and Q8R residues in *Tm*), ^Δ3R^*Tm::ZmL* (deletion of R25, R26, and R27 residues in *Tm::ZmL*), and ^3G/3R^*Tm::ZmL* (substitution of G78R, G79R, and G80R residues in *Tm::ZmL*) (Figure 1).

Compared to the WT, vector control (VC), and null siblings, all four transgenic *C. sativa* lines expressing ^SSQ/3R^*Tm* exhibited significant increases in lipid content (mg/seed) (Table 1). These lines also outperformed the unmodified *Tm* DGAT1. Among the ^SSQ/3R^*Tm* lines, one line displayed significant increases in both seed size and lipid levels, while another showed only a significant increase in seed size. The remaining two lines had a significant increase in the lipid percentage, resulting in a higher lipid/seed value (mg).

Consistent with our previous findings [35], the chimeric *Tm::ZmL* construct significantly increased seed size and lipid/seed values (mg) compared to WT, VC, and null siblings. Interestingly, the deletion of the N-terminal R motif in ^Δ3R^*Tm::ZmL* reduced both seed size and lipid content relative to the unmodified *Tm::ZmL*, bringing these parameters to levels similar to those of WT, VC, and null siblings. However, the additional R motifs further downstream of the N terminus (^3G/3R^*Tm::ZmL*) did not improve seed size or lipid/seed values (mg) compared to the unmodified *Tm::ZmL*, with seed size reduced in two out of three lines.

### 2.2. The Impact of the N-Terminal di-R Motif on the Accumulation of DGAT1 in Yeast

The findings with respect to the expression of di-R mutated DGAT1 constructs in *C. sativa* seeds suggest a potential regulatory role for the N-terminal 3R residues in Tm DGAT1. To further investigate this, we utilized yeast cells with a quadruple-mutant preventing it from being able to synthesize TAG [41]. We generated two mutants—^3R/3G^*Tm* and ^3R/3G^*Tm::ZmL*—by substituting residues R25, R26, and R27 with glycine (G25, G26, and G27) (Figure 1). Additionally, to explore the potential role of the N-terminal di-R motif interacting with oleosin during TAG biosynthesis in the ER and LD budding, we co-expressed *Tm::ZmL* (or ^3R/3G^*Tm::ZmL*) with *C. sativa* oleosin (CsOLE). These constructs were designated as *Tm::ZmL*+ and ^3R/3G^*Tm::ZmL*+, respectively.

**Figure 2:**
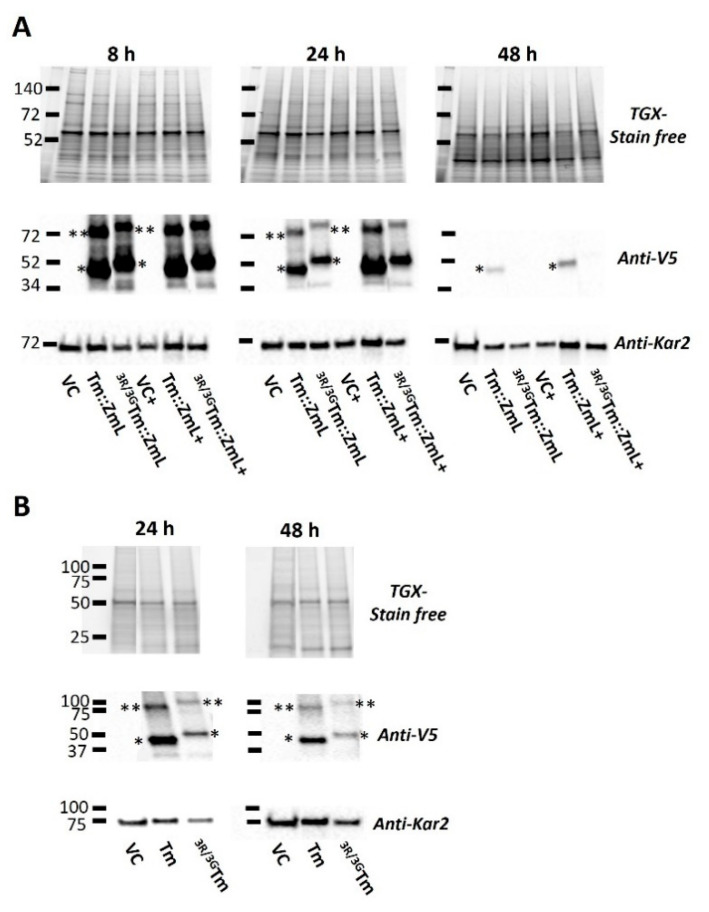
Immunoblot analysis of recombinant DGAT1s in *Saccharomyces cerevisiae*. The figure displays an immunoblot analysis conducted on microsomal proteins extracted from *S. cerevisiae* cultures at different time points: 8, 24, and 48 h. Gel loading was meticulously controlled based on equal amounts of total microsomal protein, as demonstrated by the Tris-Glycine eXtended (TGX) stain-free gel in the top panel. Quantities of the Kar2 reference protein [44] are indicated by the ~75 kDa band at the bottom membrane of each immunoblot. Recombinant DGAT1 monomers are denoted by single black asterisks. The expected monomeric sizes (in kDa) are as follows: (**A**) Tm::ZmL (62.8) and ^3R/3G^Tm::ZmL (62.5); (**B**) Tm (62.6) and ^3R/3G^Tm (62.3). Dimerized DGAT1s are marked with double black asterisks. VC stands for vector control. *VC*+, *Tm::ZmL*+, and ^3R/3G^T*m::ZmL*+ signify co-expression of DGAT1 with recombinant *Camelina sativa* oleosin. It is worth noting that the recombinant DGAT1s migrated 15–20% faster in SDS-PAGE than their predicted molecular weights. Additionally, the substitution of R25G, R26G, and R27G resulted in a slightly smaller protein size of 0.3 kDa. However, both ^3R/3G^Tm::ZmL and ^3R/3G^Tm migrated slower than their corresponding native forms. This phenomenon, referred to as “gel shifting” by Rath et al. (2009), is common for membrane proteins due to their non-polar/polar nature [45,46].

**Figure 3:**
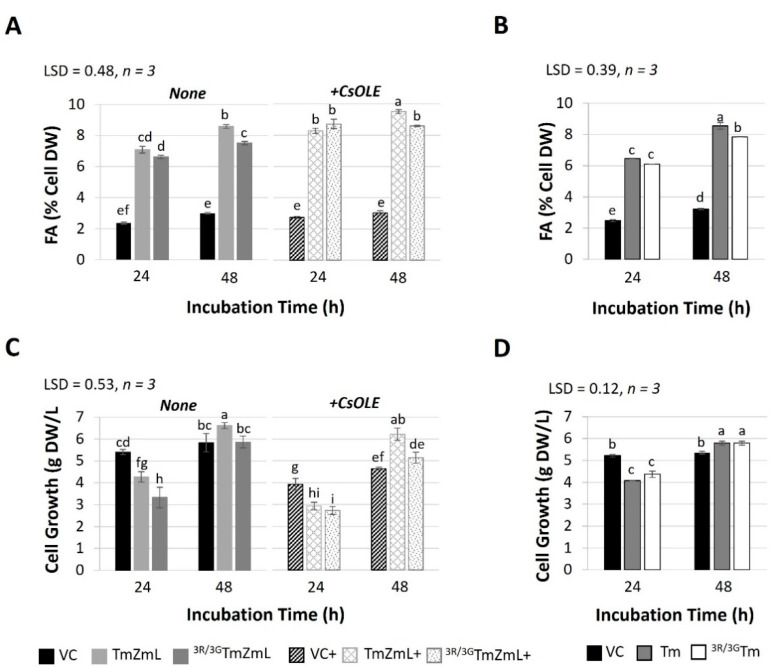
Influence of expressing *Tropaeolum majus* DGAT1 (Tm), chimeric *T. majus* and *Zea mays* long-form DGAT1s (Tm::ZmL), and di-arginine mutated DGAT1s (^3R/3G^Tm::ZmL, and ^3R/3G^Tm) in *Saccharomyces cerevisiae* quadruple mutant strain H1246 [41] on fatty acid (FA) content (**A**,**B**) expressed as a percentage of cell dry weight (DW) and cell growth (**C**,**D**) at 24 h and 48 h. VC+, Tm::ZmL+, and ^3R/3G^Tm::ZmL+ indicate co-expression with recombinant *Camelina sativa* oleosin. The data presented in the figure are expressed as means ± standard error. Different groups are distinguished by alphabet labels (a–i) that indicate significant differences (*p* < 0.05, least significant difference (LSD) test) among the groups.

Consistent with our previous findings [35], we observed a decline in the levels of recombinant Tm::ZmL and Tm during the stationary phase (Figure 2), likely due to the use of the *GAL1* promoter in *S. cerevisiae* [42,43]. However, the substitution of di-R motifs in both ^3R/3G^*Tm::ZmL* and ^3R/3G^*Tm* led to a further reduction in the long-term accumulation of recombinant proteins (Figure 2). This reduction was observed in both the absence and presence of CsOLE for ^3R/3G^*Tm::ZmL* (Figure 2A). Although no difference in FA levels was detected between the mutant and the parental counterpart in 24 h cells, a small but significant decrease in FA content was observed in 48 h cells for both ^3R/3G^*Tm::ZmL* and ^3R/3G^*Tm* compared to their non-mutate parents (Figure 3A,B). The increase in FA accumulation was observed in both non-mutated and mutated *Tm::ZmL* in the presence of CsOLE (Figure 3A).

Immunoblot analysis of crude cell extracts revealed significant differences in DGAT1 stability between the two constructs. At 24 h, band intensity quantification showed that ^3R/3G^Tm::ZmL exhibited higher fragmentation rates (24.43%) than Tm::ZmL (19.49%). This difference became more pronounced at 48 h, with the fragmentation of Tm::ZmL at 27.56% and that of ^3R/3G^Tm::ZmL at 42.26% (Supplementary Figure S1 and Supplementary Table S1). The fragment bands, detected through anti-V5 probing, appeared as smaller, discrete, immunoreactive bands corresponding to C-terminal DGAT1 fragments. These likely represent protein degradation intermediates.

Interestingly, the di-R motif substitution appeared to hinder the growth of ^3R/3G^*Tm::ZmL* yeast cells in both the absence and presence of *CsOLE* compared to the *Tm::ZmL* (Figure 3C). However, the growth of ^3R/3G^*Tm* yeast cells after 24 h and 48 h incubation periods was similar to that of *Tm* (Figure 3D). The presence of *CsOLE* also impaired the growth of VC+, *Tm::ZmL*+, and ^3R/3G^*Tm::ZmL*+ cells under both 24 h and 48 h incubation (Figure 3C).

### 2.3. Peptide Analysis of the N-Terminal DGAT1

A detailed peptide analysis of the N-terminal Tm DGAT1 derived from constructs expressed in Camelina and *S. cerevisiae* is presented in Table 2. The table contains physiochemical parameters including the length from the N-terminus to the acyl-CoA binding domain (Figure 1, amino acid); molecular weight (kDa); isoelectric point (pI); charge at pH 7; and percentages of basic, charged, and hydrophobic amino acids. These metrics are critical for understanding the biochemical properties of the peptides, which influence their function, stability, and interactions.

### 2.4. Disruption of the N-Terminal di-R Motif Within DGAT1 Reduces the Association of Oleosin with LDs

**Figure 4:**
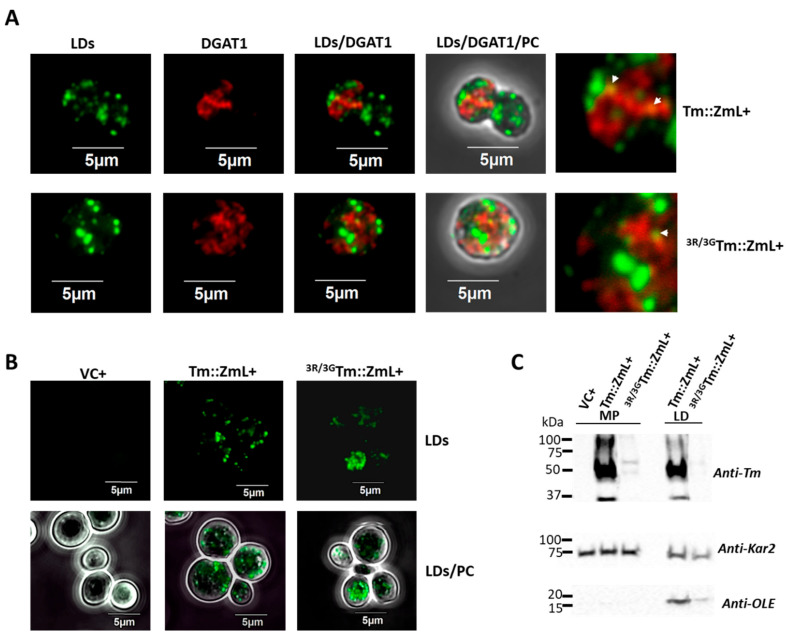
Co-localization analysis of recombinant DGAT1 and lipid droplets in *S. cerevisiae*. (**A**) Lipid droplets (LDs) in individual cells were visualized using HCS LipidTOX™ green neutral lipid stain. Recombinant DGAT1s and Tm::ZmL and ^3R/3G^Tm::ZmL immunofluorescence signals were detected by probing with the anti-Tm antibody after pre-treatment with Triton X-100 membrane-permeabilizing detergent. Co-localization of LDs and DGAT1 was observed, including using phase contrast (PC) imaging. The white arrows indicate DGAT1 localization on LDs. (**B**) LDs in individual cells of VC+, Tm::ZmL+, and ^3R/3G^Tm::ZmL+ were visualized using HCS LipidTOX™ green neutral lipid stain. (**C**) Immunoblot analysis (top panel) demonstrated that both recombinant DGAT1s were present in microsomes (MP) and LDs. Similar results were observed when detecting Kar2, the ER marker reference. Co-expression with CsOLE (Tm::ZmL+ and ^3R/3G^Tm::ZmL+) showed that CsOLE was predominantly located in LDs, with a small portion also observed in microsomes. The substitutional mutation of the N-terminal di-arginine motif of Tm reduced the level of LD-associated CsOLE, coupled with the larger-sized and less detached LDs observed in ^3R/3G^*Tm::ZmL*+ cells compared to *Tm::ZmL*+ cells (**A**,**B**).

Building on previous findings that a portion of plant DGAT1 is localized on LDs and there is a potential regulatory role for the N terminus [35], we investigated whether the triple-R motif in the N terminus of Tm is involved in this process. To confirm that some DGAT1 localizes on the LDs, we performed an immunofluorescence assay on yeast cells expressing ^3R/3G^*Tm::ZmL*+ and compared them to *Tm::ZmL*+ cells. Merged fluorescence imaging incorporating lipid staining and DGAT1 localization demonstrated a low level of DGAT1 was co-localized with LDs in both ^3R/3G^*Tm::ZmL*+ and *Tm::ZmL*+ yeast cells (Figure 4A). In addition, the ^3R/3G^*Tm::ZmL* was more dispersed compared to *Tm::ZmL*, and the LDs in ^3R/3G^*Tm::ZmL*+ cells were less uniform in size than the Tm::ZmL+ cells. However, differences in cell-cycle stages (e.g., *Tm::ZmL*+ cells underwent mitosis, while ^3R/3G^*Tm::ZmL*+ cells remained single) cannot be discounted. Lipid staining using HCS LipidTox™ also showed the variable distribution of LD sizes in ^3R/3G^*Tm::ZmL*+ cells compared to those in the *Tm::ZmL*+ cells (Figure 4B).

Immunoblot analysis of microsomes and purified LDs from yeast cultures (grown for 48 h post induction to maximize the LD yield) confirmed the presence of both Tm::ZmL+ and ^3R/3G^Tm::ZmL+ in LDs (Figure 4C). Consistent with previous findings [35], yeast karyogamy protein Kar2, a homologue of ER chaperone Bip [47], was also detected in LDs. As expected, CsOLE was predominantly localized to LDs, with a small fraction detected in microsomes (Figure 4C). Notably, a decrease in recombinant CsOLE associated with LDs was observed in ^3R/3G^*Tm::ZmL*+ cells compared to the *Tm::ZmL*+ cells.

### 2.5. The ^25,26,27^R Residues in T. majus DGAT1 Do Not Appear to Be Critical for Oligomerization

**Figure 5:**
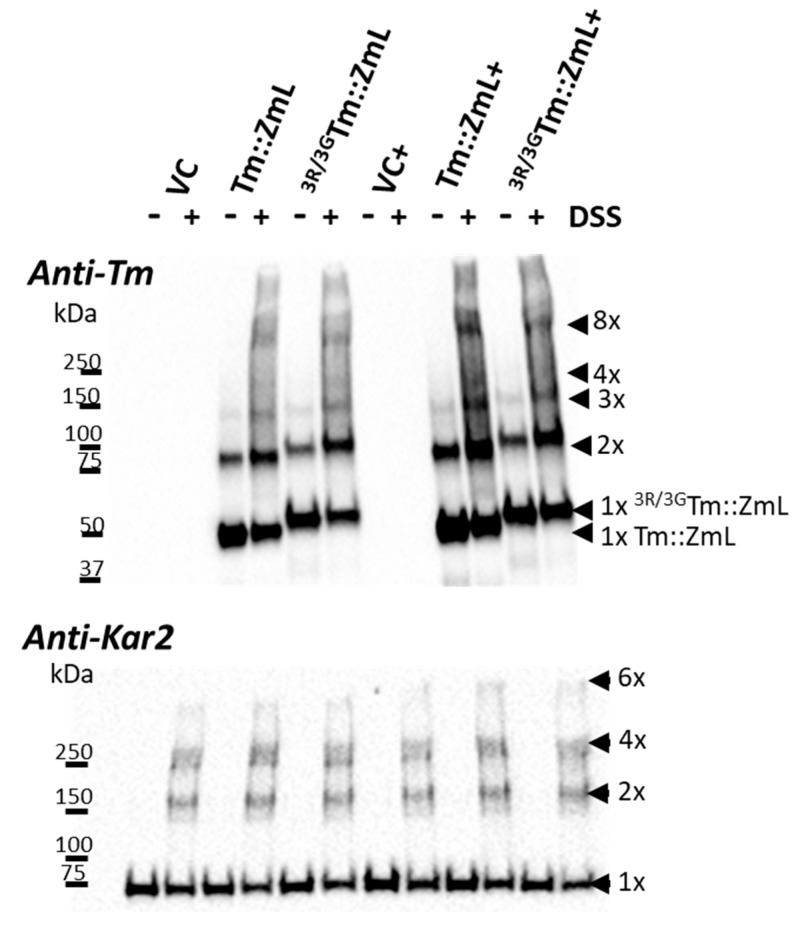
A comparison of DGAT1 oligomerization between chimeric Tm::ZmL and its N-terminal di-R motif substitution. Immunoblot analysis was conducted on microsomal proteins extracted from 8 h cultures of *S. cerevisiae* expressing various constructs: vector control (VC), Tm::ZmL, the N-terminal di-R motif-substituted mutation of ^3R/3G^Tm::ZmL, Tm::ZmL co-expressed with *Camelina sativa* oleosin (Tm::ZmL+), ^3R/3G^Tm::ZmL+, and VC+. Each microsomal preparation was divided into two aliquots, with one half treated with cross-linking agent disuccinimidyl suberate (DSS) to induce the formation of higher oligomers. The addition of DSS led to the generation of higher-order oligomers. The immunoblot analysis revealed that Tm::ZmL and ^3R/3G^Tm::ZmL exhibited similar levels of oligomerization when treated with DSS. The upper panel of the figure depicts samples with an equal loading of recombinant DGAT1 probed with the anti-N-terminal peptide sequence of Tm antibody, while the lower panel shows samples with an equal loading of microsomal proteins probed with anti-Kar2 antibody.

To maximize the recombinant DGAT1 levels, we used microsomes prepared from 8 h yeast cell cultures. In the absence of the cross-linking agent disuccinimidyl suberate (DSS), both the Tm::ZmL and ^3R/3G^Tm::ZmL (with or without CsOLE) predominantly exhibited monomeric and dimeric forms (Figure 5, top panel). Following the DSS treatment, there was a reduction in the proportion of monomeric forms and an increase in the proportions of dimers and higher oligomers. Notably, no obvious differences in the levels of oligomerization were observed between Tm::ZmL and ^3R/3G^Tm::ZmL (with or without CsOLE). Immunoblot analysis with anti-Kar2 demonstrated that equivalent levels of microsomal proteins were used in the oligomerization experiments (Figure 5, lower panel).

### 2.6. The T. majus DGAT1 ^25,26,27^R Residues May Serve as an ER Retrieval Signal and/or Aid in Protein Retention in the ER

**Figure 6:**
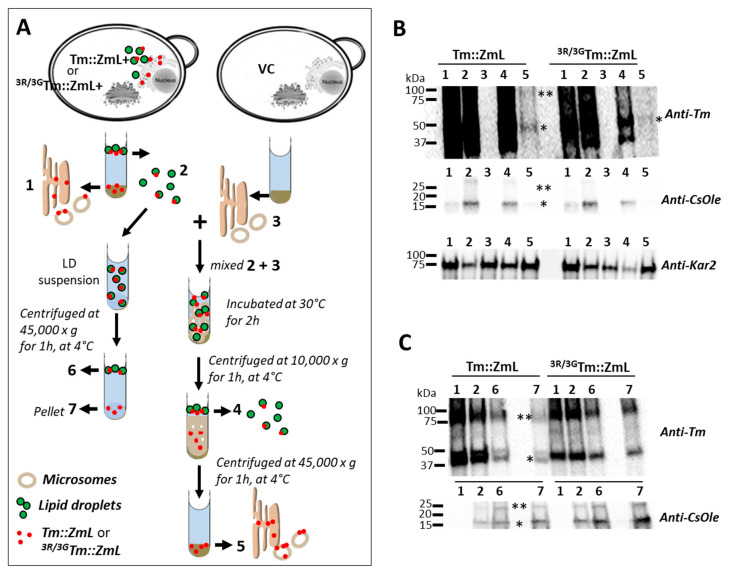
A schematic diagram outlining the in vitro experimental procedures employed to investigate the ER retrieval and lipid droplet retention of recombinant DGAT1s and oleosin in *S. cerevisiae.* (**A**) Yeast quadruple-mutant H1246 cells [41] were utilized, co-expressing different constructs: a vector control (VC) and a chimeric *T. majus* and *Zea mays* long-form DGAT1 (*Tm::ZmL*) or a di-R substituted mutant ^3R/3G^*Tm::ZmL*, along with recombinant *C. sativa* oleosin (Tm::ZmL+, and ^3R/3G^Tm::ZmL+). Cellular extraction, followed by centrifugation, resulted in separation into microsomes (1) and lipid droplets (LDs, 2). It should be noted that cells expressing VC had no LDs. VC microsomes (3) were mixed with LDs from Tm::ZmL+ or ^3R/3G^Tm::ZmL+ (2 + 3). The DGAT1 was recovered in a two-step centrifugation process that separated LDs (Lane 4) and VC microsomes (Lane 5). In a separate experiment, LDs of *Tm::ZmL*+ and ^3R/3G^*Tm::ZmL*+ underwent high-speed centrifugation to assess pellets (Lane 7). (**B**) The immunoblot probed with anti-Tm antibody (top panel) revealed the presence of recombinant Tm::ZmL and approximately 3-fold less ^3R/3G^Tm::ZmL (Table S1) with equal loading of both microsomal proteins (Lane 1) and LD-associated proteins (Lane 2). No recombinant DGAT1 was detected in the VC microsomes (Lane 3). Recombinant DGAT1 was consistently detected in the recovered LDs (Lane 4) and in the retrieved microsomes of VC (Lane 5). The two bottom panels show immunoblot analysis after probing with anti-CsOLE and anti-Kar2 antibodies, validating the adjustment for an equivalent setup of reactions for both oleosin and microsomal proteins. (**C**) The immunoblot analysis (top panel) revealed the presence of both recombinant Tm::ZmL and ^3R/3G^Tm::ZmL in both microsomes (1) and LDs (2), as confirmed by probing with anti-Tm antibody. The remaining recombinant DGAT1 was detected in the recovered LDs (6), and the recombinant DGAT1s were observed in the pellet (6). The bottom panels show the immunoblot analysis with anti-CsOLE, validating the adjustment for an equivalent setup of reactions (2, 6) and the amount of CsOLE detected in the pellet (7). Any protein-associated LDs that LDs ruptured would be in the pellet. Monomerized and dimerized DGAT1s and oleosin are marked with single and double black asterisks, respectively.

Both Tm::ZmL and ^3R/3G^Tm::ZmL accumulated to similar levels in yeast microsomes at 8 h and had comparable lipid production per cell dry weight at 24 h (Figure 2A and Figure 3A). However, at 48 h, there were noticeable decreases of recombinant ^3R/3G^Tm and ^3R/3G^Tm::ZmL and significant decreases in TAG relative to cells expressing DGATs with native di-arginine motifs. Using a cell-free system, we investigated whether the ^25,26,27^R motif is involved in ER retrieval and/or retention in the ER. A schematic overview of the experimental setup is presented in Figure 6A (see Materials and Methods for further details).

Immunoblot analysis (Figure 6B, Upper panel) of equal microsomal and LD-associated protein loads (Lanes 1, 2, and 4) revealed that ^3R/3G^Tm::ZmL levels in microsomes and LDs were approximately three-fold lower than those of Tm::ZmL at 36–40 h (Supplementary Table S2). To normalize for recombinant DGAT1 protein levels between constructs, retrieval reactions used a three-fold higher concentration of ^3R/3G^Tm::ZmL LDs to achieve equivalent DGAT1 quantities when co-incubated with VC microsomes (Lane 3). Adjustments for equal loadings of oleosin (anti-CsOLE) and microsomal (anti-Kar2) proteins were confirmed (Figure 6B, Lower panels). Following retrieval, both Tm::ZmL and the mutant were translocated from LDs to VC microsomes (Figure 6B, Upper panel, Lane 5).

While this approach ensures comparable DGAT1 protein levels for functional comparison, it simultaneously increases the total LD protein concentration in reactions containing ^3R/3G^Tm::ZmL, which exhibited comparatively reduced efficiency. This altered stoichiometry may potentially influence retrieval efficiency, as the higher concentration of non-DGAT1 LD proteins could affect membrane dynamics or compete for binding sites during the retrieval process. Therefore, differences in retrieval efficiency between Tm::ZmL and ^3R/3G^Tm::ZmL should be interpreted with consideration of these experimental constraints.

To assess DGAT1 retention on LDs, LD suspensions containing equivalent Tm::ZmL and ^3R/3G^Tm::ZmL (Figure 6C, upper panel, Lane 2) were subjected to high-speed centrifugation. Immunoblot analysis confirmed equal DGAT1 and CsOLE loadings (Figure 6C, upper and lower panels, Lane 2). After centrifugation, Tm::ZmL remained predominantly in the LD fraction, whereas ^3R/3G^Tm::ZmL was more abundant in the pellet (Figure 6C, upper panel, Lanes 6 and 7). CsOLE levels were also higher in the ^3R/3G^Tm::ZmL pellet (Figure 6C, upper panel, Lane 7), implying that the di-R mutation of DGAT1 may destabilize the oleosin–LD association more broadly.

## 3. Discussion

This study has highlighted the importance of cytoplasmic N-terminal di-R motifs in plant DGAT1s, and we demonstrated that their position is important and that they positively influence DGAT1 stability, as well as lipid accumulation and lipid droplet morphology. We explored several potential mechanisms to explain the latter.

Modifications to the di-arginine motifs of DGAT1s and their subsequent transgenic expression in the seeds of *Camelina sativa* demonstrate that not only are the motifs important but so, too, is their position relative to the N terminus. Additional motifs engineered very close to the N terminus significantly increased lipid accumulation above the unmodified version, whereas additional motifs engineered between the two endogenous motifs had little impact. In contrast, deletion of the native N-terminal motif reduced the lipid levels down to approximately WT and VC.

Immunoblot analysis (Figure 2) results from *S. cerevisiae* expressing recombinant Tm::ZmL and ^3R/3G^Tm::ZmL suggest that the N-terminal di-R motif is critical for DGAT1 stability and function, with direct consequences for yeast cell growth retardation (Figure 3). The elevated levels of C-terminal fragments detected in ^3R/3G^Tm::ZmL whole-cell extracts (Table S1) suggest defective subcellular targeting, a phenomenon known to impair *S. cerevisiae* growth through cellular stress responses and increased metabolic burden [47,48]. This mistargeting hypothesis is further supported by the time-dependent accumulation of protein fragments, indicating progressive proteolytic degradation of mislocalized DGAT1.

However, the growth kinetics of ^3R/3G^Tm yeast cells at 24 h and 48 h time points remained comparable to those of Tm cells. Whether the observed effects in Tm::ZmL result from protein mistargeting, structural alterations in the chimeric C-terminal ZmL DGAT1 domains, or the presence of CsOLE remains unresolved. Given that mistargeting of di-R mutated DGAT1 to the ER would not be significantly disruptive, the chimeric C-terminal ZmL DGAT1 domains likely become inappropriately embedded in various membranes. This speculation is particularly intriguing, as it may indicate functional differences in the capacity of modified N-terminal di-arginine motifs to interact with C-terminal domains. Additional investigations are needed to elucidate these mechanisms and establish the precise relationship between DGAT1 subcellular localization and cellular fitness.

The amino acid composition varies significantly across constructs due to the di-R mutations, with notable changes in the percentages of basic, charged, and hydrophobic residues (Table 2). The increased proportion of negatively charged residues may enhance protein solubility or promote electrostatic interactions with substrates and cofactors [49,50]. However, while hydrophobic regions are essential for lipid binding and membrane integration [51], the enhanced negative charge at the N terminus resulting from the 3R mutation may disrupt proper ER targeting [52], ultimately leading to proteolytic degradation of the mislocalized DGAT1 protein [53]. This proposed mechanism is consistent with the observed percentage increase in DGAT1 fragmentation in *^3R/3G^Tm::ZmL* crude extracts.

Consistent with prior reports [35], in yeast, we found that neither the chimeric Tm::ZmL DGAT1 nor its di-R mutant is strictly confined to the ER and both can associate with LDs, possibly via ER–LD membrane contact sites (MCS) during LD budding [54]. Given the phospholipid structure of the LDs (monolayer), we assume that associated DGAT1 is likely non-functional or only partial functional. However, functionality could exist if the DGAT1 were present in a raft of the ER bilayer in the LD. The location of ER marker Kar2 [44] in LDs supports the idea that under certain conditions (such as ER remodeling or lipid imbalance) ER-resident proteins (e.g., DGAT1) can become transiently associated with LDs, but this is not considered their functional or stable localization. Notably, Kar2 enrichment in LDs is not merely due to ER contamination but occurs under ER stress [55], and that Kar2 degradation proceeds via a microautophagy pathway independent of core autophagy components, implicating LDs in ER proteostasis [56].

In higher eukaryotes, LDs are enveloped by abundant scaffold proteins such as oleosins in plants and perilipins in animals [57,58]. Although *S. cerevisiae* lacks homologous proteins, Jacquier et al. (2013) demonstrated that heterologously expressed oleosins correctly localize to LDs in yeast. In the absence of LDs, these oleosins localize to the ER bilayer, where they undergo degradation [59]. This concurs with our finding that CsOLE was undetectable when expressed in cells that do not generate LDs but was present in microsomal and LD fractions of cells co-expressing either *Tm::ZmL* or ^3R/3G^*Tm::ZmL* (Figure 4C).

The reduced LD-associated CsOLE levels in ^3R/3G^*Tm::ZmL*+, combined with the formation of larger and fewer detached LDs (Figure 4), suggest that di-arginine mutations in the DGAT1 N terminus interfere with proper LD formation. These observations are consistent with findings by Wilfling et al. (2013), who identified two distinct LD subpopulations in Drosophila and mammalian cells: smaller LDs of relatively constant size and larger LDs that grow through association with ER-to-LD translocated enzymes involved in TAG biosynthesis, including glycerol-3-phosphate acyltransferase 4 and DGAT2 [60]. This parallel suggests that plant DGAT1 may play a similar role in determining LD morphology. Future investigations should examine whether the N-terminal di-R mutations disrupt this regulatory mechanism and elucidate the underlying molecular basis for such disruption.

Immunofluorescence imaging revealed that Tm::ZmL produced uniformly sized LDs, whereas ^3R/3G^Tm::ZmL resulted in irregularly shaped LDs (Figure 4A,B), suggesting altered LD budding or stability, potentially indicating that the mutated DGAT1 is not properly retained in the ER. This retention defect likely impairs normal LD budding, possibly through steric hindrance. However, additional factors may contribute to this morphology, including insufficient oleosin coverage of enlarged LDs or altered DGAT1 distribution, leading to inefficient oleosin association [61].

Oligomerization is a common feature of membrane proteins; it influences their enzymatic activity, stability, and subcellular localization [62,63]. N-terminal truncation of DGAT1 in both *Homo sapiens* and *Brassica napus* disrupts dimer formation [64,65], supporting the hypothesis that DGAT1 oligomerization enhances ER retention and limits dissociation with LDs. Interestingly, in *Mus musculus* and long-form DGAT1 from *Zea mays*, similar truncations reduce higher-order oligomers without fully inhibiting dimerization [30,35]. Although di-R motifs are known to mediate hetero-oligomerization [39,40], our results indicate that the conserved 25R, 26R, 27R motif is not required for Tm DGAT1 oligomerization (Figure 5). This is consistent with the structure of DGAT1, where the dimer interface is separated from the N-terminal sequences [66]. However, DSS is a random cross linker of primary amines and will cross link peptides in close proximity [67]. As such, the similar in vitro oligomerization patterns seen with Tm::ZmL and ^3R/3G^Tm::ZmL indicates that their distribution and proximity to more recombinant protein are similar. However, it does not mean that these form oligomers in the cell.

Protein localization is largely determined by peptide-encoded signals [68]. These include retrieval motifs that direct ER and Golgi membrane proteins back to their original compartments [36,69]. Di-R motifs, particularly those at the cytoplasmic N terminus, play a central role in retrieving ER membrane proteins from the Golgi or ER–Golgi intermediate compartment [38]. Importantly, the presence of multiple retrieval motifs enhances the efficiency of ER targeting [36]. The present study asked whether plant DGAT1 localized in LDs could be retrieved to the ER and whether the conserved N-terminal di-R motifs contribute to this process. Our observations of Tm::ZmL and ^3R/3G^Tm::ZmL trafficking from LDs to the microsomes of VC cells suggest that LD-to-ER retrieval is possible (Figure 6). This DGAT1 retrieval may serve to maintain ER protein homeostasis or prevent interference with oleosin packing on LDs [61]. However, the homeostatic function would only be beneficial if DGAT1 is retrieved intact and correctly folded or if removing DGAT1 added to LD stability [51,53].

**Figure 7:**
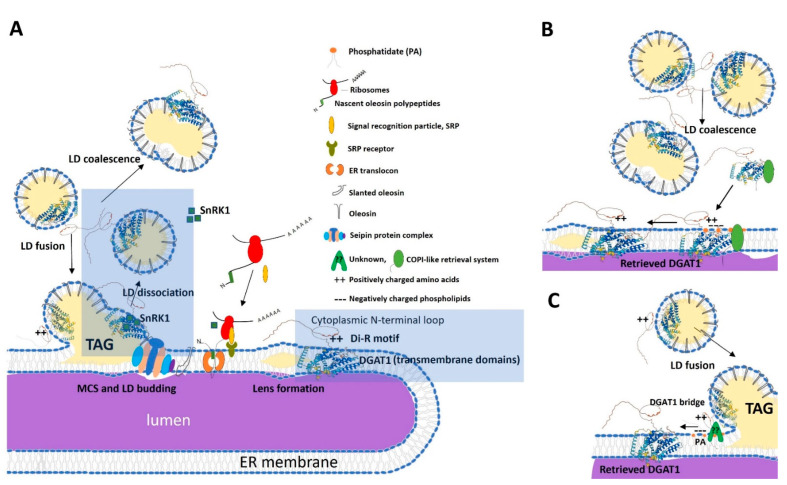
Lipid droplet (LD) biogenesis at the LD–ER membrane and DGAT1 retrieval mechanism at the LD–ER membrane contact site (MCS). (**A**) During lens-structure formation, triacylglycerol (TAG) synthesized by diacylglycerol acyltransferase 1 (DGAT1) accumulates between the leaflets of the ER bilayer. DGAT1 is localized to the ER membrane and consists of a cytoplasmic N-terminal loop containing a conserved di-arginine (R) motif and nine putative transmembrane domains [35]. The signal recognition particle (SRP) co-translationally targets oleosin, which contains multiple ER-targeting sequences in its hydrophobic domain [68], to the ER. The nascent oleosin polypeptide chain is translocated through the translocon into the ER lumen [72,73]. LD budding at the LD–ER MCS during LD biogenesis requires ER-localized seipin protein complexes [71]. LDs are separated from the ER and released to the cytosol with associated oleosin, as well as improperly folded DGAT1. We proposed that phosphatidate (PA) and sucrose non-fermenting related kinase 1 (SnRK1) modulate DGAT1 phosphorylation (highlighted blue rectangle), which may determine the DGAT1’s membrane topology, dependent on the lipid environments (such as the LD phospholipid membrane and ER phospholipid bilayer) [31,74,75]. This dynamic rearrangement of DGAT1 topology allows for intraorganellar switching between LDs and the ER. Furthermore, LDs may coalesce with others or fuse with developing LDs on the ER membrane [58], creating a potential mechanism for DGAT1 retrieval. (**B**) Proposed retrieval mechanism: Coalesced LDs release a phosphorylated, less active form of DGAT1 into the cytosol, where PA lipid signaling and unknown tethering proteins (e.g., seipin, coatomer protein complex, and endomembrane vesicle trafficking cargo complex) recruit and mediate DGAT1 retrieval to the ER. (**C**) Alternative mechanism: Positively charged amino acid-rich segments (arginine and lysine) in the cytoplasmic N-terminal DGAT1 interact with negatively charged membrane phospholipids of the ER, potentially aided by unknown tethering proteins [76].

Understanding this process requires consideration of LD biogenesis. During LD formation, LDs remain in continuity with the ER through MCS. DGAT1-mediated TAG accumulates between the ER bilayer leaflets, eventually forming lens-like structures that bud off as LDs (Figure 7A). Oleosin, which contains multiple ER-targeting signals in its hydrophobic domain, is co-translationally targeted to the ER via the signal recognition particle (SRP) pathway [70,71]. Proteins such as seipins, localized at ER-LD MCS, are required for proper LD biogenesis and detachment [54].

The topology of membrane proteins is influenced by the surrounding lipid environment. Post-translational modifications, including phosphorylation of positively charged extramembrane domains, can alter topology [77,78]. In *B. napus*, DGAT1 activity is regulated through two key mechanisms: phosphatidic acid (PA) binding and phosphorylation via SnRK1 kinase. Notably, SnRK1 translocates between the nucleus and ER in response to cellular energy status, creating a dynamic regulatory system [74,79]. It would be particularly interesting to determine if DGAT1 regulation by PA and SnRK1 also influences its trafficking between LDs and the ER and whether the impaired trafficking observed with ^3R/3G^Tm::ZmL disrupts this regulatory network. Loss of the N-terminal di-R motif may compromise any of the interactions discussed and subsequently disrupt the ER–LD MCS, lens formation, and TAG packaging into LDs [80].

Immature LDs can merge with each other or with nascent LDs still associated with the ER (Figure 7A). Phosphorylated DGAT1 (in its less active form) may be released from coalescing LDs into the cytosol, where it may be recruited back to the ER via lipid signaling (e.g., PA) and unknown tethering factors (Figure 7B). Alternatively, the DGAT1 recycling mechanism occurs when mature LDs fused to developing LDs at the ER membrane. Here, the positively charged N-terminal residues (R and K) may interact electrostatically with negatively charged phospholipid head groups of the ER membrane [72,76]. If this were the case, then substitution of the 3R motif would reduce the electrostatic attraction and, therefore, impair ER retrieval. Additionally, protein tethering complexes such as seipins, coatomer complexes, and vesicle trafficking machinery may facilitate LD–ER cargo transfer [73,75]. While arginine-to-lysine substitutions could further dissect the role of positive charge, prior work indicates that di-lysine motifs functionally mimic di-R in ER retrieval [38,40]. Our glycine substitution data (Figure 2, Figure 3, Figure 4, Figure 5 and Figure 6) demonstrate that charge loss—not arginine-specific properties—is the primary driver of DGAT1 destabilization and mislocalization. Thus, the conserved di-R motif likely operates through electrostatic interactions with ER membranes or tethering proteins, consistent with its role in dynamic LD–ER trafficking.

## 4. Materials and Methods

### 4.1. The di-R Mutated DGAT1 Expression Vectors for C. sativa

We designed three constructs with di-R mutations in DGAT1 as follows: ^SSQ/3R^*Tm*, which involved substituting S6R, S7R, and S8R residues in Tm; ^∆3R^*Tm::ZmL*, where the R25, R26, and R27 residues were deleted in Tm::ZmL; and ^3G/3R^*Tm::ZmL*, which substituted G78R, G79R, and G80R residues in Tm::ZmL (Figure 1).

To simplify the construction of expression cassettes, we ensured that these mutated N-terminal coding sequences contained an *Xba* I site at the 5′ UTR and an *Xho* I site within the coding sequence of the conserved residues (L-S-S) in the middle of the acyl-CoA binding domain (Figure 1). The mutated coding sequence was synthesized by GENEART and subcloned to replace the native N-terminal sequences of Tm and Tm::ZmL in pDONR™ 221, as previously described by Winichayakul et al. [35]. Subsequently, these di-R mutated DGAT1 constructs were inserted into the pBR2 binary vector using GATEWAY^®^ LR cloning (Thermo Fisher Scientific, Waltham, MA, USA). The pBR2 vector was previously designed to regulate the expression of recombinant DGAT1 under the control of the *Brassica napus* 1.7 S storage promoter [35,81].

### 4.2. Camelina Experiments

The growth conditions, transformation, and selection procedures, as well as lipid extraction and analysis methods, were carried out in accordance with the protocols established by Winichayakul et al. [35].

### 4.3. The di-R Mutated DGAT1 Expression Vectors for S. cerevisiae

The di-R mutation of Tm DGAT1 involved substituting R25G, R26G, and R27G residues, as shown in Figure 1. For the ease of expression cassette construction, we designed the N-terminal coding sequences of ^3R/3G^Tm to incorporate an *Eco* RI site in the 5′ UTR and an *Xho* I site within the coding sequence of the conserved residues (L-S-S) in the acyl-CoA binding domain (Figure 1). The mutated sequence was synthesized by GENEART and subcloned into the pYES2.1/V5-His-TOPO cloning vector (Thermo Fisher Scientific, Waltham, MA, USA) to replace the native N-terminal sequences of full-length Tm and the chimeric Tm::ZmL, resulting in the generation of ^3R/3G^*Tm* and ^3R/3G^*Tm::ZmL* constructs (Supplementary Figure S2A).

To identify fractionation of LDs and microsomes from the *S. cerevisiae* cell extract, we generated constructs for co-expression of DGAT1 and a specific LD-associated protein, CsOLE (Xm_010450170, NCBI, Bethesda, MD, USA). An inducible system containing galactose promoters GAL1 and GAL10 in the yeast expression vector (K4150-01, Life Technologies, Waltham, MA, USA) was utilized, as previously described by Winichayakul et al. [6]. Tm::ZmL or ^3R/3G^Tm::ZmL constructs were cloned by exchanging the *Eco* RI/*Xba* I digested fragment in the pYES2.1 vector containing the *Arabidopsis thaliana* DGAT1:V5:6xHis cassette (Supplementary Figure S2B). The coding sequence of the CsOLE (XP_010448472) was optimized for expression in *S. cerevisiae* by GENEART, and the optimized version, flanked by *Avr* II/*Sac* II restriction sites at the 5′ end and a stop codon, replaced the oleosin 0-0 ORF.

### 4.4. Yeast Experiments

The growth conditions, transformation protocols, crude protein extraction methods, microsome protein preparation procedures, immunoblot analysis techniques, immunolocalization of recombinant DGAT1, and in vitro cross-linking assays were performed in accordance with the methods described in the study conducted by Winichayakul et al. [35].

### 4.5. Lipid Droplet Staining

Yeast spheroplasts were prepared following the protocol outlined by Winichayakul et al. [35] and fixed onto polylysine-coated slides. To stain neutral lipids, LipidTOX™ Green neutral lipid stain (H34475, Invitrogen™, Carlsbad, CA, USA) was diluted 1:200 in 1× phosphate-buffered saline (1 × PBS). The diluted stain was added to the fixed spheroplasts, ensuring complete coverage; then, a coverslip was placed over the sample. The fluorescence of stained LDs was visualized using confocal microscopy with appropriate filter sets for the detection of Alexa Fluor^®^ 488 dye.

### 4.6. In Vitro Study of ER Retrieval of DGAT1s from LDs

Approximately 5 g (fresh weight, FW) of yeast cells cultured for 36–40 h expressing VC, *Tm::ZmL*+, and ^3R/3G^*Tm::ZmL*+ cells were thoroughly washed in 10–50 mL of MilliQ H_2_O containing 1 mM phenylmethylsulfonyl fluoride. The cells were then resuspended in half the cell volume of extraction buffer (EB) consisting of 1xPBS, 0.6 M sucrose, protease inhibitor cocktails (P8215, 1 mL/20 g FW, Sigma-Aldrich, Auckland, New Zealand), and lyticase (L2524, 5000 U/5 g FW, Sigma-Aldrich, Auckland, New Zealand).

The cell suspension underwent a 90 min incubation at 30 °C on a slow-speed rotary shaker to facilitate cell lysis. To further disrupt the cells, the suspension underwent vortexing at speed level 6 (L46, Labinco B.V., Breda, The Netherlands) for 1 min using acid-washed glass beads (425–600 μm, G8772, Sigma-Aldrich, Auckland, New Zealand) at a similar volume to the cells, followed by a 1 min interval on ice. This vortexing step was repeated three times. The cell crude extract was then centrifuged at 1000× *g* for 10 min at 4 °C to remove cell debris. The supernatant was collected and subjected to a second centrifugation step at 10,000× *g* for 1 h at 4 °C. This step aimed to separate the LDs from the supernatant, and as expected, no LDs were observed in the VC cells. The supernatant obtained at this step was ultracentrifuged at 45,000× *g* (Rmin, 2.45 cm) for 1 h at 4 °C to collect the microsomes.

To create the necessary buffering components for ER retrieval, the supernatant from the final step of the VC cell extract served as the retrieval reaction (RR) buffer. LDs and microsomes were individually resuspended in 100 μL of RR buffer, and the total protein content was determined. For the RR conditions, 100 μg of LD proteins from *Tm::ZmL*+ or 300 μg of LD proteins from ^3R/3G^*Tm::ZmL*+ was mixed with 150 μg of microsomal proteins from VC. The reaction volume was adjusted to 0.25 mL with RR buffer and incubated at 30 °C for 2 h with intermittent shaking (2 min of shaking followed by a 10 min rest cycle). The RR samples were then subjected to centrifugation at 10,000× *g* for 30 min at 4 °C to separate the LDs from the reaction. The supernatant obtained at this step was collected and further ultracentrifuged at 45,000× *g* for 1 h at 4 °C to collect the microsomes. Both recovered LDs and microsomes were washed with the EB and resuspended in 50 μL of EB for LDs and 100 μL of EB for microsomes. Protein samples (0.5 mg/mL) were prepared according to the methods described by Winichayakul et al. [6]. Unless indicated, 5 μg of protein was loaded onto an SDS-PAGE gel for subsequent immunoblot analysis [6,35].

### 4.7. In Vitro Study of LD Retention of DGAT1 and Oleosin

In a separate experiment, yeast cell culture and protein extraction were conducted following the procedures outlined earlier. Washed LDs obtained from the 10,000× *g* centrifugation, comprising 100 μg of LD proteins from *Tm::ZmL*+ or 300 μg of LD proteins from ^3R/3G^*Tm::ZmL*+, were subjected to high-speed centrifugation at 45,000× *g* for 1 h at 4 °C to isolate the pellet. The resulting pellet was resuspended in 50 μL of EB and subsequently prepared for protein loading, following the procedures mentioned earlier.

### 4.8. Immunoblot Analyses

Protein samples were separated by SDS-PAGE on 4–15% gradient polyacrylamide gels and transferred to PVDF membranes (Bio-Rad Trans-blot Turbo system). Immunoblotting was performed as described by Winichayakul et al. (2013) [6]. PVDF membranes were incubated with antibodies using the following manufacturers and dilutions: mouse anti-V5 (R96025, Life Technologies, Waltham, MA, USA), 1:10000; rabbit anti-Kar2 (sc33630, Santa Cruz Biotech, Dallas, TX, USA), 1:2500; rabbit anti-Tm (customized antibody, GenScript, Piscataway, NJ, USA), 1:2500; rabbit anti-CsOLE (customized antibody, GenScript, Piscataway, NJ, USA), 1:2500; anti-mouse IgG-HRP (626520, Life Technologies, Waltham, MA, USA), 1:5000; anti-rabbit IgG-HRP (A6154, Sigma-Aldrich, Auckland, New Zealand). Protein–antibody complexes were visualized using Advansta Western Bright ECL spray (K12049-D50, Advansta Inc., San Jose, CA, USA) and ChemiDoc MP Imager (Bio-Rad, Auckland, New Zealand).

We assume that the anti-Tm antibody produced in rabbit against the N-terminal Tm DGAT1 fragment has similar sensitivity for detecting both Tm::ZmL and ^3R/3G^Tm::ZmL, as the cross-reacted DGAT1 band intensities in the 8 h culture microsomal extracts were comparable (Figure 2A).

### 4.9. Statistical Analysis

The experimental data were analyzed using statistical tests in RStudio version 3.6.0. Student’s two-tailed *t*-test was used for pairwise comparisons, while one-way or two-way ANOVA was employed for multiple comparisons. The ANOVA models included the fixed effect of DGAT1s and their corresponding controls. To determine significant differences among treatment means, Bartlett’s test was performed to assess the homogeneity of variances, and the Shapiro–Wilk normality test was used to check the normal distribution of the data. *p*-values were adjusted using the BH method [82] to control the false discovery rate. The reported results include means, standard errors (SE), and least significant difference (LSD) values. Fixed effects were considered significant if the corresponding *p*-value was less than 0.05 (*p* < 0.05).

## 5. Conclusions

This study elucidates the critical role of conserved N-terminal di-R motifs in the stability and function of plant DGAT1, a key enzyme in TAG biosynthesis. Through targeted mutagenesis of DGAT1 in *Tropaeolum majus*, we demonstrated that disruption of these motifs significantly reduces lipid accumulation in both *Camelina sativa* seeds and *Saccharomyces cerevisiae* cells. The di-R motifs were found to be essential for maintaining DGAT1 stability, proper LD organization, and the retention of oleosin on LDs.

Immunofluorescence and immunoblot analyses revealed that DGAT1 localizes to LDs and the ER, with the di-R motifs facilitating retrieval of DGAT1 from LDs back to the ER. This dynamic trafficking suggests a regulatory mechanism for DGAT1 activity and lipid homeostasis. This study highlights the potential of these motifs in engineering oil content in plants and microorganisms, offering insights for biotechnological applications aimed at enhancing lipid production.

In summary, the conserved N-terminal di-R motifs in DGAT1 are pivotal for enzyme stability, lipid accumulation, and LD dynamics. These findings advance our understanding of TAG biosynthesis and provide a foundation for future research into the optimization of oil yield in agricultural and industrial contexts.

## Figures and Tables

**Table 1:** *Camelina sativa* seed analysis.

Plants *n* = 6	HOM Seed Size (mg/Seed)	±SE	Null Seed Size (mg/Seed)	±SE	HOM Seed Lipid (%)	±SE	Null Seed Lipid (%)	±SE	HOM Seed Lipid (mg/Seed)	±SE	Null Seed Lipid (mg/Seed)	±SE
**WT**	1.17	0.024			27.30	0.67			0.29	0.009		
**VC**	1.17	0.026			25.96	0.63			0.28	0.007		
**Tm#2**	1.15	0.045	1.15	0.036	29.58 *	0.58	26.21	0.92	0.32 *	0.009	0.27 27	0.008
**^SSQ/3R^Tm#8**	1.32 **^#^	0.034	1.16	0.032	28.97 *	1.29	24.39	0.88	0.38 **^##^	0.018	0.28	0.014
**^SSQ/3R^Tm#13**	1.37 **^##^	0.048	1.18	0.034	27.78	1.09	27.45	0.59	0.37 **^###^	0.008	0.32	0.014
**^SSQ/3R^Tm#30**	1.24	0.027	1.18	0.032	28.91 *	0.43	27.94	0.60	0.35 *^##^	0.011	0.32	0.010
**^SSQ/3R^Tm#38**	1.27	0.029	1.17	0.018	30.56 *	1.18	26.52	0.40	0.38 *^##^	0.020	0.31	0.008
**Tm::ZmL#13**	1.29 **	0.022	1.12	0.029	31.66 ***	0.25	25.43	1.00	0.41 ***	0.010	0.29	0.017
**^Δ3R^Tm::ZmL#1**	1.16 ^##^	0.039	1.21	0.036	25.52 ^##^	1.26	25.78	0.82	0.30 ^##^	0.021	0.31	0.016
**^Δ3R^Tm::ZmL#15**	1.18 ^##^	0.011	1.16	0.030	28.10 ^#^	1.17	26.87	0.48	0.33 ^##^	0.013	0.31	0.004
**^Δ3R^Tm::ZmL #21**	1.24 ^#^	0.054	1.19	0.044	26.70 ^##^	1.15	25.30	1.29	0.33 ^##^	0.013	0.30	0.004
**^3G/3R^Tm::ZmL #1**	1.30 ***	0.020	1.20	0.023	30.60 ***	1.34	23.36	1.41	0.40 ***	0.023	0.29	0.017
**^3G/3R^Tm::ZmL #20**	1.15 ^#^	0.017	1.16	0.044	31.55 ***	0.74	27.49	0.36	0.36 **^#^	0.006	0.32	0.013
**^3G/3R^Tm::ZmL #21**	1.14 ^#^	0.024	1.18	0.013	31.41 ***	1.39	27.30	0.89	0.36 **^#^	0.021	0.32	0.009

Comparison of weight and lipid content in seeds of wild-type (WT), vector control (VC), and individual homozygous (HOM) lines of a full-length or a chimeric DGAT1 with/without di-Arg (R) mutation and their respective null sibling. Significant differences relative to the WT, VC, and null siblings indicated by *, **, and *** represent *p* < 0.05, *p* < 0.01, and *p* < 0.001, respectively; significant differences relative to the non-mutated parent indicated by ^#^, ^##^, and ^###^ represent *p* < 0.05, *p* < 0.01, and *p* < 0.001, respectively. SE is the standard error value of the means.

**Table 2:** Report of protein statistics of the N-terminal Tm DGAT1 peptides.

Construct	Length (aa)	MW (kDa)	Isoelectric Point	Charge at pH 7	% Basic	% Charged	% Hydrophobic
Tm and Tm::ZmL	121	12.90	6.37	−0.78	13.2	24.8	43.8
*Camelina sativa*							
^SSQ/3R^Tm	121	13.06	9.96	2.22	15.7	27.3	43.8
^∆3R^Tm::ZmL	118	12.43	5.01	−3.78	11.0	22.9	44.9
^3G/3R^Tm::ZmL	121	13.19	9.96	2.22	15.7	27.3	41.3
*Saccharomyces cerevisiae*							
^3R/3G^Tm and ^3R/3G^Tm::ZmL	121	12.60	5.01	−3.78	10.7	22.3	46.3

Data reported by Geneious Prime version 2019.1.1.

## Data Availability

The original contributions presented in the study are included in the article and Supplementary Materials. Further inquiries can be directed to the corresponding author.

## Supplementary Materials

The following supporting information can be downloaded at: https://www.mdpi.com/article/10.3390/ijms26157406/s1.
