# Supplementary material for: A Conserved N-Terminal Di-Arginine Motif Stabilizes Plant DGAT1 and Modulates Lipid Droplet Organization
Source: Int J Mol Sci. 2025 Jul 31;26(15):7406. doi: 10.3390/ijms26157406 (PMC12347784; doi:10.3390/ijms26157406)
Supplement: Supplementary file 1 [file ijms-26-07406-s001.zip › ijms-3714899-supplementary.pdf]

Table S1 Semi-quantitation of recombinant DGAT1s and their derivative fragments from yeast crude extract and protein immunoblotting.

DGAT1	Cultivation	Relative Front	Volume Intensity Unit	Identification	% of Fragmentation
**Tm::ZmL**	24 h	0.036	3,868,666	Oligomers	19.49%
		0.335	22,415,744	Dimer	
		0.527	29,659,418	Monomer	
		0.781	4,549,208	~22 kDa C-terminal	
		0.884	2,490,026	~15 kDa C-terminal	
		0.968	6,505,904	~ 10 kDa C-terminal	
**$^{3R/3G}$Tm::ZmL**	24 h	0.039	1,053,338	Oligomers	24.43%
		0.309	13,830,404	Dimer	
		0.480	18,572,348	Monomer	
		0.776	3,376,794	~22 kDa C-terminal	
		0.886	1,413,676	~15 kDa C-terminal	
		0.959	6,023,760	~ 10 kDa C-terminal	
**Tm::ZmL**	48 h	0.053	6,419,206	Oligomers	27.56%
		0.322	12,057,567	Dimer	
		0.513	21,276,745	Monomer	
		0.756	4,716,068	~22 kDa C-terminal	
		0.854	3,896,273	~15 kDa C-terminal	
		0.955	6,510,931	~ 10 kDa C-terminal	
**$^{3R/3G}$Tm::ZmL**	48 h	0.057	1,237,731	Oligomers	42.26%
		0.294	14,834,835	Dimer	
		0.479	12,179,475	Monomer	
		0.760	4,930,080	~22 kDa C-terminal	
		0.851	3,622,080	~15 kDa C-terminal	
		0.954	12,123,720	~ 10 kDa C-terminal	

Table S2 Semi-quantitation of recombinant DGAT1s from protein immunoblotting.

DGAT1	Organelle Proteins	Relative front	Volume Intensity Unit	Volume Intensity Ratio Tm::ZmL/$^{3R/3G}$Tm::ZmL
Tm::ZmL	Microsomes (1)	0.641	3,710,390	3.02
$^{3R/3G}$Tm::ZmL	Microsomes (1)	0.615	1,228,251	
Tm::ZmL	Lipid droplets (2)	0.644	4,149,505	3.03
$^{3R/3G}$Tm::ZmL	Lipid droplets (2)	0.618	1,370,448	

The intensity of protein bands in the immunoblotting image from Figure 6B (anti-Tm) was accessed by analyzing the volume intensity using Software Image Lab version 5.2.1 (Bio-Rad).

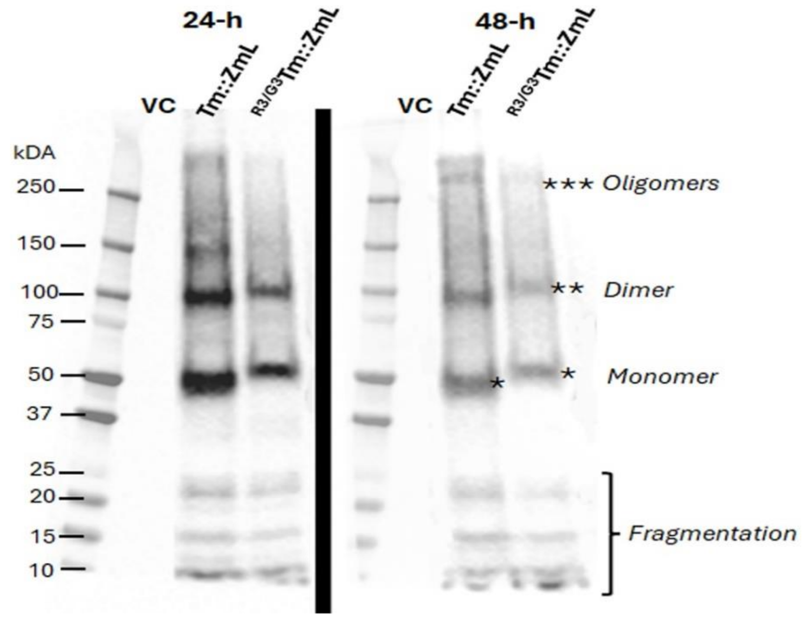

Figure S1. Immunoblot analysis of recombinant DGAT1 expression and fragmentation in *S. cerevisiae*.

Crude proteins were extracted from *S. cerevisiae* cultures at 24- and 48-hours post-induction, with gel loading normalized to 10 µg total protein per lane.

Single black asterisks indicate recombinant DGAT1 monomers with expected molecular weights of 62.8 kDa (Tm::ZmL) and 62.5 kDa ($^{3R/3G}$Tm::ZmL). Double and triple black asterisks mark DGAT1 dimers and higher-order oligomers, respectively. VC represents the vector control.

Anti-V5 antibody probing revealed small, discrete immunoreactive bands corresponding to C-terminal DGAT1 fragments, likely representing protein degradation intermediates. Protein band intensities were quantified using ImageLab software version 5.2.1 (Bio-Rad), with results presented in Table S2.

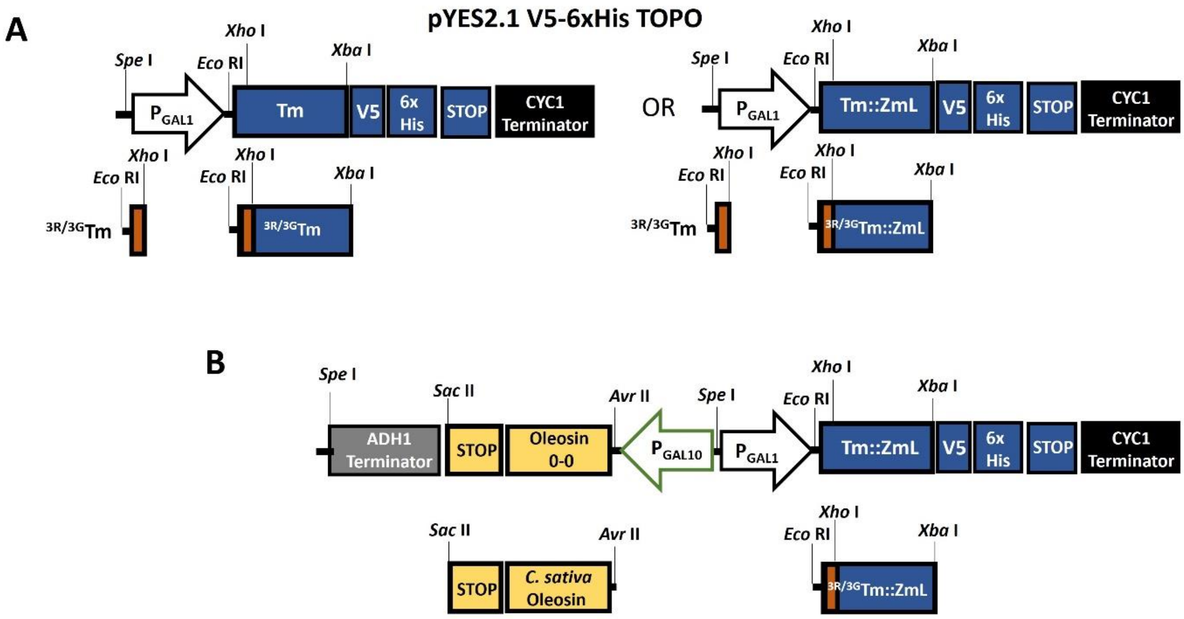

Figure S2. Construct designs for co-expression of site-specific di-arginine mutated N-terminal sequences of DGAT1 and oleosin in *Saccharomyces cerevisiae*.

(A) The Di-arginine mutation of *T. majus* DGAT1 (Tm) (indicated by the orange-brown box) involved substituting the residues R25G, R26G, and R27G. The sequence was designed to incorporate an *Eco* RI site in the 5' UTR, and an *Xho* I site within the coding sequence for the conserved residues L-S-S in the acyl-CoA binding domain (Figure 1). The mutated sequence was synthesized by GENEART and subcloned to replace the native N-terminal sequences of the full-length Tm and the chimeric Tm and *Zea mays* long-form (*Tm::ZmL*) constructs (indicated by the blue box) in the pYES 2.1 V5-6x histidine TOPO cloning vector (Life Technologies, K4150-01). This process resulted in the generation of the constructs $^{3R/3G}$*Tm* and $^{3R/3G}$*Tm::ZmL*.

(B) Our inducible system, previously described by Winichayakul et al. (2013), contains galactose promoters *GAL1* and *GAL10* in the pYES2.1/V5-His-TOPO yeast expression vector [6]. In this system, *Tm::ZmL* or $^{3R/3G}$*Tm::ZmL* were cloned by replacing the *Eco* RI/*Xba* I digested fragment in the expression cassette of this vector. The *Camelina sativa* oleosin (XP_010448472) ORF was optimized for expression in *S. cerevisiae* by GENEART, with *Avr* II/*Sac* II restriction sites flanking at the 5' end and a STOP codon, and it replaced the oleosin 0-0 ORF (indicated by the yellow box).
